# Ten years of IRCAD, Barretos, SP, Brazil

**DOI:** 10.1590/acb370608

**Published:** 2022-09-19

**Authors:** Eduardo Crema, Armando Geraldo Franchini Melani, Luís Gustavo Capochin Romagnolo, Jacques Marescaux

**Affiliations:** 1 PhD, full professor. Universidade Federal do Triângulo Mineiro – Division of Digestive Tract Surgery – Uberaba (MG), Brazil.; 2 MSc, technical and scientific director. IRCAD Latin America, and physician at Americas Integrated Oncology Center – Rio de Janeiro (RJ), Brazil.; 3 MD. Hospital de Câncer de Barretos – Pio XII Foundation, and scientific coordinator, IRCAD Latin America – Barretos (SP), Brazil.; 4 MD, founder and scientific coordinator. IRCAD Latin America – Barretos (SP), Brazil.

**Keywords:** Minimally Invasive Surgical Procedures, Mentoring, Technology

## Abstract

Minimally invasive surgery represented a significant milestone in modern surgery; however, continuous innovation and the emergence of new technologies pose new challenges in terms of surgical learning curves since new interventions are associated with increased surgical complexity and a higher risk of complications. For this reason, surgeons are aware of the beneficial effects of “learning before doing” and the importance of safely implementing new surgical procedures in order to obtain better patient outcomes. Considered the largest Latin American training center in minimally invasive surgery, IRCAD Barretos, São Paulo, Brazil, makes it possible to acquire surgical skills through training in different and the most complex areas of medicine, providing the experience of real and simulated situations, with focus on innovation. The center possesses state-of-the-art infrastructure and technology, with a very high-level teaching staff and an affectionate and hospitable reception. Since its inauguration, in 2011, the center has already qualified numerous professionals and has placed the country in a privileged position in terms of surgical knowledge. The present article describes the activities developed over these ten years of the institute in Brazil as the largest training center for surgeons of the continent in order to address the importance of surgical skills training.

## Introduction

Institut de Recherche contre les Cancers de l’Appareil Digestif (IRCAD) was founded by the French doctor and professor Jacques Marescaux in 1994 at the university hospital of Strasbourg, France, with the aim of creating projects and actions in the area of healthcare technologies by combining medical and imaging techniques, thus integrating an innovation system of excellence for training and specialization^1^. The success of the initiative has revolutionized the teaching of minimally invasive surgery. In 2010, IRCAD expanded its operations to Taiwan, in Asia, and also opened two units in Africa, in Lebanon (2019) and in Rwanda (2021), and a unit in Wuxy, China (2021)^2^.

The first IRCAD unit in Brazil, that comprises an area of 7.200 square meters, was inaugurated on July 9, 2011 in the city of Barretos, SP, through a partnership with the Pio XII Foundation, which maintains and is responsible for the Cancer Hospital, now called Hospital de Amor, the largest cancer center in Latin America^3^. The efforts of the president of Hospital de Amor de Barretos, Henrique Prata, of Dr. Armando Melani and of Professor Marescaux consolidated IRCAD Barretos in Brazil, which is nowadays the largest Latin American training center in minimally invasive surgery. The center provides excellence in education and possesses a highly qualified teaching staff formed by Brazilian experts and experts from different countries. The center offers validated training programs, including revised and improved managements and practices, advanced technology and real-time transmission of the procedures performed, thus enabling surgeons of different nationalities to acquire high-level skills, and placing Brazil in a privileged position in the world in terms of surgical knowledge. Realization of the project was also possible due to the contributions of state governments and the federal government, as well as the companies Covidien and Karl Storz, leaders in the market of surgical and endoscopic instruments. In 2017, a second center was opened in the country in the city of Rio de Janeiro, RJ^4^.

The advent of minimally invasive surgery represented a significant milestone in modern surgery and has become a new surgical doctrine^5^. At IRCAD, the technology combined with practical teaching supervised by renowned experts allows tactical and technological training, promoting benefits for thousands of surgical cases across the continent. The laparoscopic surgery training laboratory was implemented to help surgeons overcome the challenging learning curve. According to Dr. Melani, more than mastering the technology, it is necessary to understand how it will benefit patients^4^.

Technical skills are only one component of surgical practice, which also requires theoretical knowledge, risk assessment and decision-making processes, communication, teamwork, cost analysis, and management. This set of knowledge and its application within an ethical context will define the skills necessary for the training of doctors graduating from medical school, as well as for the training of experts^6^. Over these 10 years of IRCAD Barretos, approximately 12,000 surgeons were trained and course assessment by the participants showed a high degree of satisfaction. Commitment to surgical education requires deep dedication from educators, as well as intense engagement of participants who need to quickly learn new techniques and be able to use them with good outcomes^7^. In view of these considerations, this article describes the activities developed by IRCAD Barretos over these 10 years, highlighting the importance of surgical skills training.

## IRCAD and the importance of surgical skills training

The use of endoscopic approaches started in gynecology, in a specular study involving pregnant women. However, it was only in the 20th century that technology advanced to the point of accessing the intra-abdominal cavity. The first laparoscopy was performed on a dog by the German surgeon Georg Kelling in 1901. In humans, the Swedish internist Hans Christian Jacobaeus performed the first laparoscopic surgery in 1911^8^. However, laparoscopy started to experience a boom by 1989, when Dr. Philippe Mouret, in Lyon, France, performed the first laparoscopic cholecystectomy. From that time on, the technique started to be considered a great advancement of digestive surgery^9^. However, according to Wolfe *et al.* 10, the emergence of minimally invasive surgery has led to an increase in the number of iatrogenic bile duct injuries, as many surgeons around the world have changed from the open surgery paradigm to these procedures without any prior training.

At the end of the second millennium, surgeons conceded to the importance of the pathophysiology of surgical trauma, attempting to improve therapeutic outcomes through minimal accesses in order to reduce trauma^11^. With the technological development and use of lenses and cameras, advances in surgery included endoscopic procedures through natural orifices or artificial openings (laparoscopic procedures) combined or not with a robotic platform that offers the same surgery in a minimally invasive manner. This technological evolution led to the development of devices that, in turn, expanded the possibilities of the technique, which changed from a diagnostic to a therapeutic procedure^12^. 

The first tool used by surgeons for training and skill acquisition in laparoscopic surgery was an inanimate model in the form of a black box. After intensive training, surgeons were able to improve the skills needed for video-assisted laparoscopic procedures. However, the main shortcoming of this simple simulator was the limited space to objectively assess student performance^13^.

The principle of minimal invasion of body integrity, *i.e.*, the least possible tissue trauma and minimal changes in physiological homeostasis, extends far beyond the anatomical approach and includes factors considered of fundamental importance for surgeons. When compared to laparotomy, the advantages are reduced postoperative pain, smaller incisions, better respiratory function, shorter hospital stay, quick return to habitual and work activities, with recovery lasting 1/3 of the time compared to conventional surgery, broad access to the entire abdominal cavity, and a better esthetic outcome, as well as significant cost reduction and a lower rate of postoperative infections, incisional hernias, and eviscerations^14^^,^ 15.

In the last two decades, surgical education has undergone profound transformations due to the revolution caused by minimally invasive surgery. The advances in laparoscopic surgery led to the use of this approach in a range of procedures, which has become the standard technique in general, gastrointestinal, gynecological, thoracic, and urological surgery^16^. However, no laparoscopic training is available in most medical schools or for surgeons interested in adopting minimally invasive techniques. This fact is the result of the absence of a specific curriculum that includes the necessary steps for effective training in laparoscopic surgery. In the era of minimally invasive procedures, especially those performed by laparoscopic surgery, psychomotor skills must not and cannot be trained directly on the patient, but must rather be acquired by surgical simulation using organic, inorganic or virtual models, which precede the phase of surgical field training in humans^17^^-^ 19.

A critical analysis of the training of residents in laparoscopic surgery in Brazil suggests that training for the acquisition of skills in medical residency programs requires a more adequate pedagogical teaching process in order to provide a more solid educational base than the current one^20^. Drossard^21^ evaluated structured surgical residency training in Germany, analyzing 10 surgical subspecialties. The authors found that training centers exist outside the university hospitals; however, the courses offered are expensive (1,550 euros), and the training is not accessible to most surgeons.

Simulation as a tool for learning psychomotor skills in minimally invasive procedures has become the new education model in surgery. First, it is necessary to know and learn how to handle the devices and instruments of laparoscopic surgery, considering the difficulties in skill acquisition in laparoscopy such as the radical change in the perceived environment, loss of depth sensation, an indirect camera image, lack of three-dimensional vision, two-dimensionality, use of long instruments introduced through a fixed point determined by the position of the trocar, and the fulcrum effect (instruments that move at a fixed point to the abdominal wall causing paradoxical inverse movements). In addition, there is clear impairment of tactile perception caused by the lack of tissue sensation. The prolonged learning curve increases the risk of complications, and special attention therefore needs to be paid to these risks. Training laparoscopic skills outside the operating room is necessary to optimize patient outcome and to minimize the occurrence of learning curve-associated complications^22^^,^ 23.

Surgeons are aware of the existence of learning curves, of the beneficial effects of learning before doing, and of the importance of safely implementing new surgical procedures. However, continuous surgical innovation poses new challenges in terms of surgical learning curves since new interventions are associated with a higher surgical complexity and lower relative efficacy compared to older procedures^24^.

In the case of esophagectomy, the beneficial effects of minimally invasive surgery have been well documented^25^^,^ 26. Previous studies on learning curves in minimally invasive esophagectomy have focused on the results that are directly related to the procedure itself, such as blood loss and operative time^27^^,^ 28. However, recent studies have shown that the learning curve results were clinically relevant, highlighting anastomotic leakage, survival, and mortality^29^. Learning-associated morbidity is a recognized problem (morbidity during a learning curve, which could have been avoided if the patient were operated on by an experienced surgeon who completed the learning curve). Surgical learning can increase patient safety and improve outcomes in the current surgical era^30^.

Anatomical knowledge, sensory perception and manual skills of the surgeon are factors frequently associated with conventional surgery. Thus, advancement of the classical approach is surgeon dependent. Laparoscopic surgery, on the other hand, encompasses a virtual environment, telesurgery and robotics, and minimally invasive surgery is therefore characterized as technology dependent. Thus, adequate theoretical-practical training is required to perform the procedure safely, and training surgeons before treating patients is essential to improve outcomes^31^. For the development of psychomotor skills, one of the most appropriate training techniques is based on the theory of Fitts and Posner, which proposes three learning stages: cognition (when the skill is learned), integration (when performance approaches the skill), and automation (when the skill has become fully automatic and can be performed without thinking too much about the task)^32^.

Sadideen and Kneebone^33^ conducted a review on the aspects of educational theories that could be applied to surgical teaching. The most important theories were:

- acquisition and retention of motor skills (Miller’s pyramid; theory of Fitts and Posner);
- development of expertise through repeated actions and reinforcement (Ericsson’s theory);
- availability of expert supervision (Vygotsky’s theory);
- learning in the environment of practice (theories of Lave and Wenger);
- feedback on learning practical skills (theories of Boud, Schon and Ende);
- affectivity in the learning process.

Simulation using virtual reality at the start of endoscopic training has emerged as a necessary addition to conventional learning. It also provides several benefits, including a low-risk environment, valuable improvement in patient safety, and optimization of endoscopy time^34^. Tejos *et al.* 35 demonstrated that surgeons trained in a virtual simulator develop greater skills in the execution of knots within a shorter period of time.

In a systematic review, Spiliotis *et al*.^36^ found that teaching laparoscopic skills in general surgery residency programs was difficult and not standardized considering that the development of motor and spatial skills is necessary, which are more difficult to acquire, and that many factors affect surgical training during medical residency. In the United States and Europe, government determinations have reduced the maximum number of weekly working hours for medical residents. Consequently, residents must achieve the same level of competence as their predecessors with fewer hours of training. In addition, the cost of teaching a surgeon inside the operating room on a patient is very high (US$ 53 million per year in the United States), generating financial pressure. Another important factor is related to the physician’s civil liability. According to recent epidemiological studies, medical errors are common causes of death in the United States. Patients exposed to inexperienced surgeons in training have led the education system to reassess issues related to medical training.

Surgeons employing minimally invasive techniques acquire surgical skills in courses in which they are trained using laparoscopic training boxes, inanimate models, virtual reality simulation, artificial organs, and cadavers, in addition to live animal models and cadaver training^37^. According to Dawe *et al.* 38, the use of human or animal cadavers for learning surgical maneuvers is becoming increasingly controversial, a fact that considerably increased the use of virtual environments as a tool for learning psychomotor skills in minimally invasive surgery and for the simulation of complete surgical procedures. More recently, robotic surgery simulation has been implemented and validated in training.

At IRCAD Barretos, the Minimally Invasive Surgery Training Center performed several exercises on the platform used for certification in robotic surgery. On this platform, surgeons have access to real simulation of all movements they can perform on the robot during surgeries, such as suturing and ultrasonic scalpels, demonstrating the importance of improving learning curves.

Beyer-Berjot *et al.* 39 evaluated the educational value of advanced training in laparoscopic abdominal surgery. The authors analyzed 54 original studies, which mainly assessed training in video-assisted gastric and colorectal procedures in porcine models. The studies showed participant satisfaction after training. However, the level of evidence was low, highlighting the importance of high-quality studies to assess the educational value of advanced training in laparoscopic abdominal surgery.

Within this context, education in the health area influenced by technological advances and by the speed of information, associated with the progress in knowledge, requires innovative teaching methodologies that provide critical and creative training of students, moving away from old methods that focus on repetition and memorization. The effective learning of psychomotor skills requires an approach that includes different components, such as the practice setting, structure of the practice, teacher/student dialogue, and pedagogical strategies^40^.

**Figure 1:**
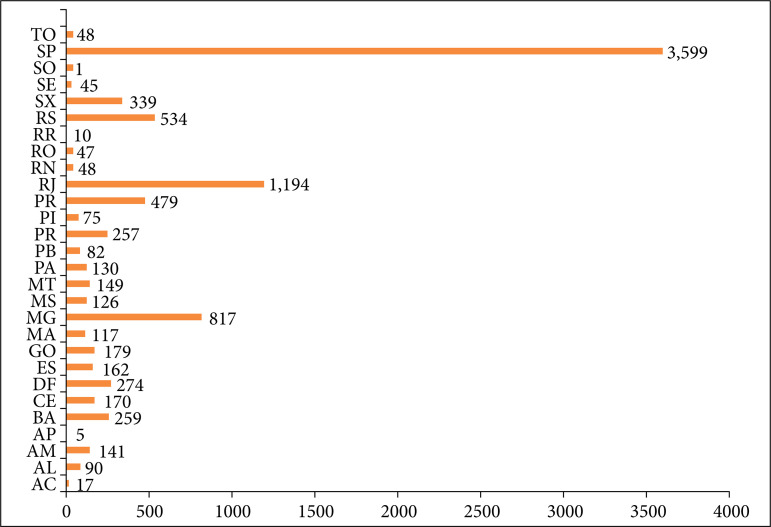
Total number of Brazilian students participating in courses at IRCADBarretos, SP, Brazil, over the 10 years and their respective states.

**Figure 2:**
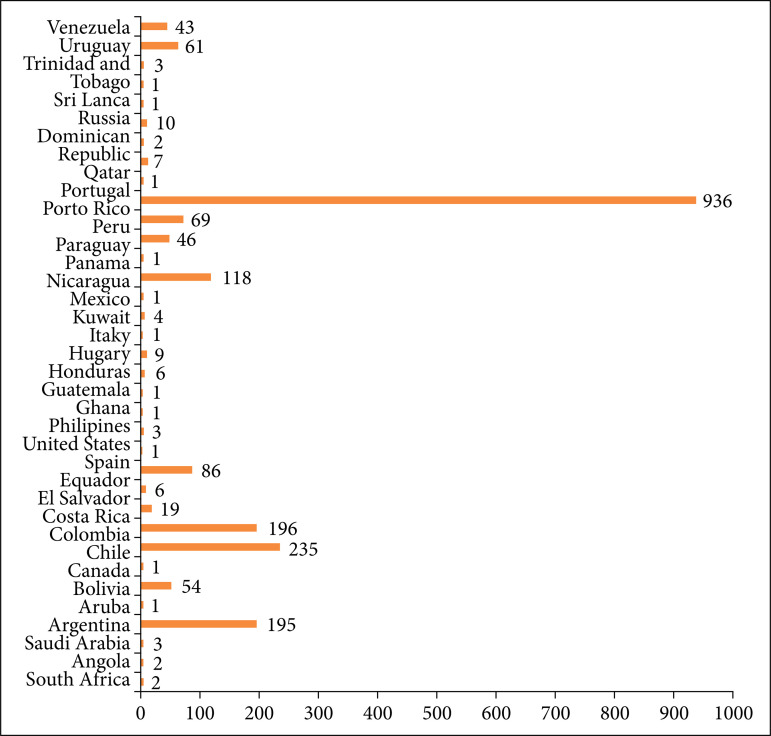
Total number of international students participating in courses at IRCADBarretos, SP, Brazil, over the 10 years and their country of origin.

**Figure 3:**
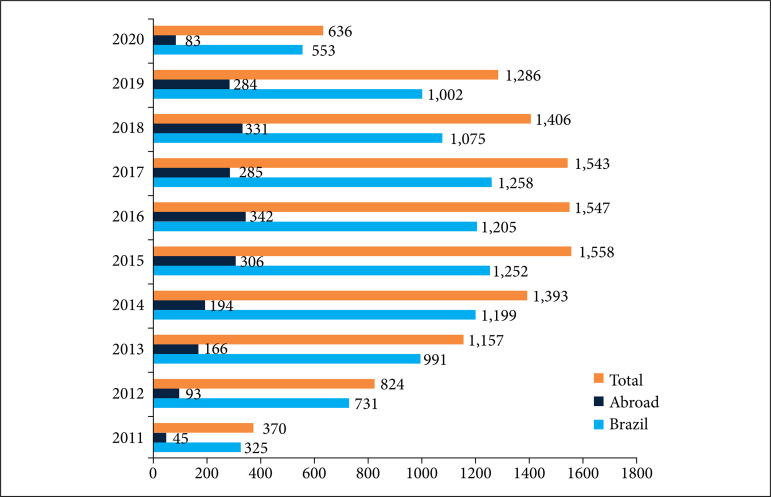
Comparison of the number of IRCAD courses taught from 2011 to 2020 in Brazil and abroad.

After the inauguration of IRCAD in Brazil, the first official course on digestive surgery was taught on July 11, 2011. In that year, an additional 17 minimally invasive surgery courses were offered in the fields of general, digestive, colorectal, bariatric, pediatric, urological, and gynecological surgery, interventional endoscopy, skull base surgery, arthroscopy, and natural orifice endoscopic transluminal surgery. Over the 10 years of operation in the city of Barretos, IRCAD contributed to the training of 11,720 students. Among them, 9,589 were Brazilian students from all states of the federation and 2,131 were foreigners from 35 countries. Among the 9,589 Brazilian students, 3,599 were from the state of São Paulo; 1,194 from Rio de Janeiro; 817 from Minas Gerais; 534 from Rio Grande do Sul; 479 from Paraná; 339 from Santa Catarina; 274 from Distrito Federal; 257 from Pernambuco; 259 from Bahia; and 117 from Maranhão, demonstrating the importance of disseminating these courses to all the 26 states of the country and Distrito Federal (Fig. 1). Among the international students who participated in the courses, 936 were from Peru; 235 from Chile; 195 from Argentina; 196 from Colombia, and 118 from Mexico (Fig. 2). Figure 3 shows an increase in the number of courses offered by IRCAD in Brazil and abroad between 2011 and 2020.

The learning of surgical techniques necessarily includes the acquisition of several psychomotor skills, which are defined as mental and motor activities that are necessary to perform a given manual task. Despite variations in assessment methods, skills, and surgical parameters, studies have reported the effectiveness of simulation training in clinical practice^41^. Simulator-trained surgeons performed surgical procedures faster and more accurately and with a lower percentage of intraoperative errors or postoperative complications than the conventionally trained group, thus minimizing the risks of iatrogenic events and offering surgeons a safe and comfortable environment without the pressure of the operating room^42^.

According Vajsbaher *et al*.^43^, it has been well documented that spatial cognition is an important factor for skill acquisition and for the performance of surgeons in minimally invasive surgery. One of these spatial cognitive skills is visuospatial ability. This skill enables the individual to develop internal mental representations of visual patterns and to use these representations to solve complex spatial problems, allowing to retain, retrieve and transform visual and spatial information according to its spatial locus. Within the context of minimally invasive surgery, these processes govern the surgeon’s ability to, for example, insert the trocars efficiently and safely, to judge the spatial relationship between the tip of the instrument and the organ, to apply appropriate force to the instrument, and to successfully perform technically challenging tasks like suturing. The authors add that a deeper understanding of the nature of these spatial skills related to minimally invasive surgery would facilitate simulation-based medical education.

According to Wahba *et al*.^44^, three-dimensional display techniques have promoted spatial orientation in minimally invasive surgery and two-dimensional 4K ultra-high definition screens can create a high-resolution image of the operative field, which may also improve surgical performance. IRCAD accompanies the major technological advances related to minimally invasive surgery using three-dimensional and 4K imaging systems equipped with cameras, lights, and the most advanced medical devices in the world. These systems are available in the 21 laparoscopic surgery stations of the experimental laboratory for surgical procedures and hands-on training, where surgeons practice the processes seen in theoretical sessions by training on live tissue, dry lab, and anatomical specimens. There is also Operating Room Number 1, which provides doctors and teams with integrated control of the devices, allowing greater attention to the patient and the procedure.

Alvarez-Lopez *et al.* 45 reported that a new low-cost portable simulator called Simulator of Minimally Invasive Surgery Mediated by Gestures-Virtual Reality (SIMISGEST-VR) proved to be suitable for training professionals with no previous experience and to enable them to learn basic psychomotor skills for performing minimally invasive surgeries. The authors also emphasized that the high cost and low availability of virtual reality simulators in surgical specialty training programs in low- and middle-income countries make it necessary to develop and validate new models of low-cost portable simulators that will allow ubiquitous learning of psychomotor skills in minimally invasive surgery.

The courses at IRCAD are divided into modules that permit the participating surgeons to separately train flexible, rigid, fluorescence and three-dimensional endoscopy, with aggregate resources being available that further improve the image quality. These resources include homogeneous illumination even in deeper areas, ensuring precision in the tasks to be performed, increased contrast especially in relation to the edges of the structures, improved sharpness at the time of dissection, and the possibility to change the tone of a certain region, ensuring visualization of the vascular part of the tissues. The technological upgrade also offers new light sources, switching from Xenon to light-emitting diode (LED) technology, a more recent technology that has a longer lifespan.

IRCAD Barretos also possesses a multimedia auditorium equipped with very high-resolution technology (4K HD). Surgical procedures are transmitted live, directly from the surgical center of Hospital de Amor de Barretos, allowing students to learn in real time laparoscopic and robotic surgeries. The work of IRCAD is currently supported by partnerships with globally recognized companies in the area such as Karl Storz, Medtronic, and the center is therefore able to offer students high-performance technologies.

The center now offers new training modalities that encompass various medical fields using supervised hands-on learning, step-by-step training with increasing complexity, and comprehensive tutoring by faculty surgeons. The most popular courses are general, digestive oncology, gynecological and hepatic surgery, endometriosis, and the general course for residents. Training is aimed at benefitting the development of the cognitive, affective, and psychomotor domains in order to improve the efficiency of health professionals, thus addressing the growing concern regarding patient and professional safety in hospital and extra-hospital settings whose origin is precisely the questioning of efficiency in health education, among other factors^46^.

At IRCAD, teachers have the habit of always being close to students during training days, whether during mealtimes offered on site or even outside, as the exchange between professionals constantly benefits the evolution of medicine, one of the greatest achievements of IRCAD as an institution^47^.

As a lecturer who teaches training classes at IRCAD, every time I go to Barretos, I am received in an affable, welcoming, and warm manner, in addition to the very high quality of all services provided, which are committed to the well-being of everyone there. The institute also offers a diversified and high-standard gastronomy, providing moments of joy, relaxation, and exchange of experiences. I am honored to be part of this journey of success, dedication, innovation, and commitment to surgical education (Eduardo Crema, *in memoriam*).

## Final considerations

Although a reality in many centers in Brazil, minimally invasive surgery is still not part of most medical residency programs, a fact that requires training outside the operating room to promote the effective learning of laparoscopic skills and to minimize the occurrence of learning curve-associated complications.

IRCAD Barretos, consolidated in Brazil as one of the most renowned minimally invasive surgery training centers, is nowadays a reference for surgical specialization infrastructure and considered one of the best and most modern training, teaching, and research centers in the world. Over the 10 years since its inauguration, the center has remained aligned with the greatest technological advances related to minimally invasive surgery, bringing together renowned national and international experts. In addition, the center provides a pleasant and welcoming environment, which facilitates the exchange of experiences and ideas. The courses taught at IRCAD, whether theoretical or practical, have a high degree of scientific excellence and cover different medical areas using supervised hands-on learning, step-by-step training, and comprehensive tutoring by experts in minimally invasive surgery. Since its inauguration, in 2011, IRCAD has contributed to the training of approximately 12,000 Brazilian and international surgeons, improving the daily surgical practice of these professionals and consequently providing benefits to patients by training highly qualified surgeons in the use of minimally invasive techniques.
